# Crystal structure of 2,2′-bis­[(2-chloro­benz­yl)­oxy]-1,1′-bi­naphthalene

**DOI:** 10.1107/S2056989015014322

**Published:** 2015-08-06

**Authors:** Rajamani Raja, Mani Jayanthi, Perumal Rajakumar, A. SubbiahPandi

**Affiliations:** a Department of Physics, Presidency College (Autonomous), Chennai 600 005, India; b Department of Organic Chemistry, University of Madras, Guindy, Chennai-25, India

**Keywords:** crystal structure, binaphth­yl, anti­microbials, anti­biotic properties, minimum toxicity, hydrogen bonding

## Abstract

In the title binaphthyl compound, C_34_H_24_Cl_2_O_2_, the dihedral angle between the two naphthyl ring systems (r.m.s. deviations = 0.016 and 0.035 Å) is 76.33 (8)°. The chloro­phenyl rings make dihedral angles of 58.15 (12) and 76.21 (13)° with the naphthyl ring to which they are linked. The dihedral angle between the planes of the two chloro­phenyl rings is 27.66 (16)°. In the crystal, C—H⋯O hydrogen bonds link mol­ecules into chains propagating along [1-10]. The chains are linked by C—H⋯π inter­actions, forming a three-dimensional framework.

## Related literature   

For the synthesis and biological activity of naphthalene compounds, see: Upadhayaya *et al.* (2010 ▸); Rokade & Sayyed (2009 ▸). For the crystal structure of a very similar compound, 4,4′-{[[1,1′-bi­naphthalene]-2,2′-diylbis(­oxy)]bis­(methyl­ene)}dibenzo­nitrile, see: Fu & Zhao (2007 ▸).
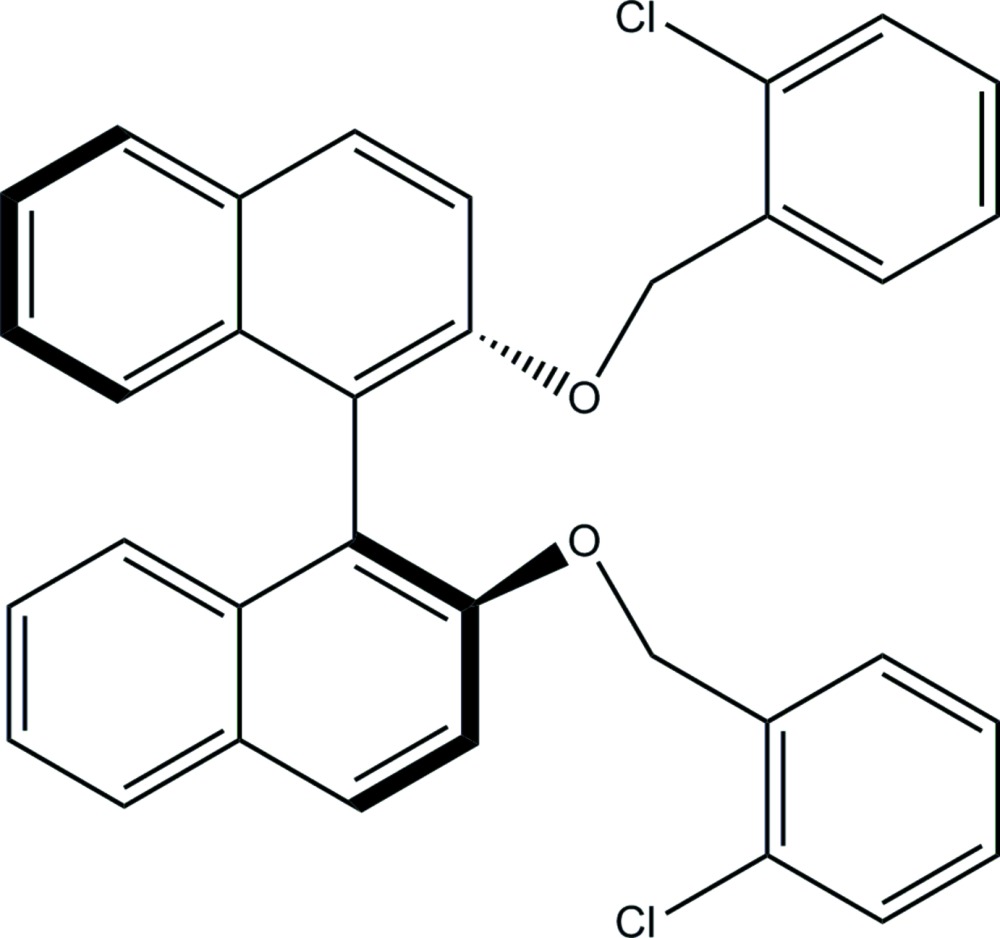


## Experimental   

### Crystal data  

C_34_H_24_Cl_2_O_2_
*M* *_r_* = 535.43
Monoclinic, 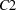

*a* = 11.1983 (3) Å
*b* = 14.6094 (4) Å
*c* = 16.3263 (4) Å
β = 92.622 (2)°
*V* = 2668.19 (12) Å^3^
*Z* = 4
Mo *K*α radiation
μ = 0.27 mm^−1^
*T* = 293 K
0.35 × 0.30 × 0.25 mm

### Data collection  

Bruker SMART APEXII CCD diffractometer
Absorption correction: multi-scan (*SADABS*; Bruker, 2008 ▸) *T*_min_ = 0.909, *T*_max_ = 0.921
10688 measured reflections
4153 independent reflections
3804 reflections with *I* > 2σ(*I*)
*R*_int_ = 0.019

### Refinement  

*R*[*F* ^2^ > 2σ(*F* ^2^)] = 0.042
*wR*(*F* ^2^) = 0.116
*S* = 1.04
4153 reflections
343 parameters
1 restraint
H-atom parameters constrained
Δρ_max_ = 0.35 e Å^−3^
Δρ_min_ = −0.47 e Å^−3^
Absolute structure: Flack (1983 ▸), 1709 (76%) Friedel pairs
Absolute structure parameter: −0.01 (8)

### 

Data collection: *APEX2* (Bruker, 2008 ▸); cell refinement: *SAINT* (Bruker, 2008 ▸); data reduction: *SAINT*; program(s) used to solve structure: *SHELXS97* (Sheldrick, 2008 ▸); program(s) used to refine structure: *SHELXL97* (Sheldrick, 2008 ▸); molecular graphics: *ORTEP-3 for Windows* (Farrugia, 2012 ▸); software used to prepare material for publication: *SHELXL97* and *PLATON* (Spek, 2009 ▸).

## Supplementary Material

Crystal structure: contains datablock(s) global, I. DOI: 10.1107/S2056989015014322/su5184sup1.cif

Structure factors: contains datablock(s) I. DOI: 10.1107/S2056989015014322/su5184Isup2.hkl

Supporting information file. DOI: 10.1107/S2056989015014322/su5184Isup3.cml

**Fig. 1.**
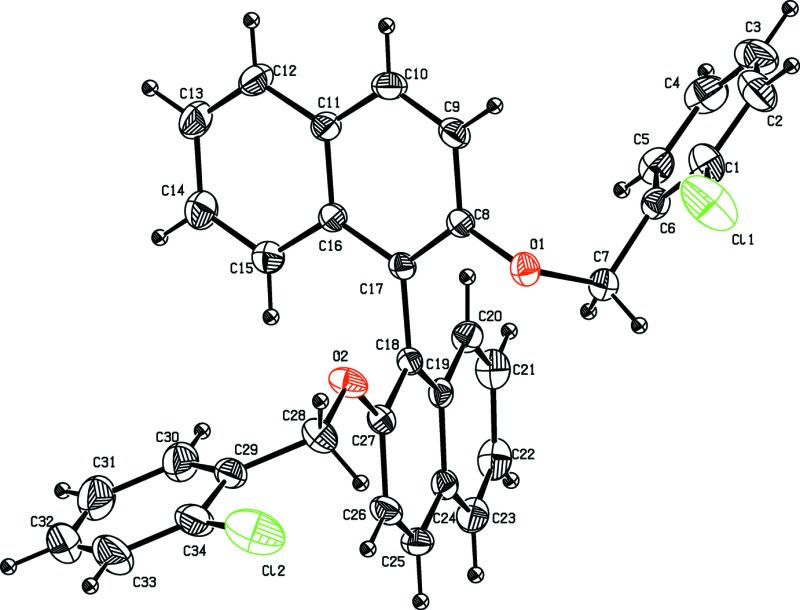
The molecular structure of the title compound, with atom labelling. Displacement ellipsoids are drawn at the 30% probability level.

**Fig. 2.**
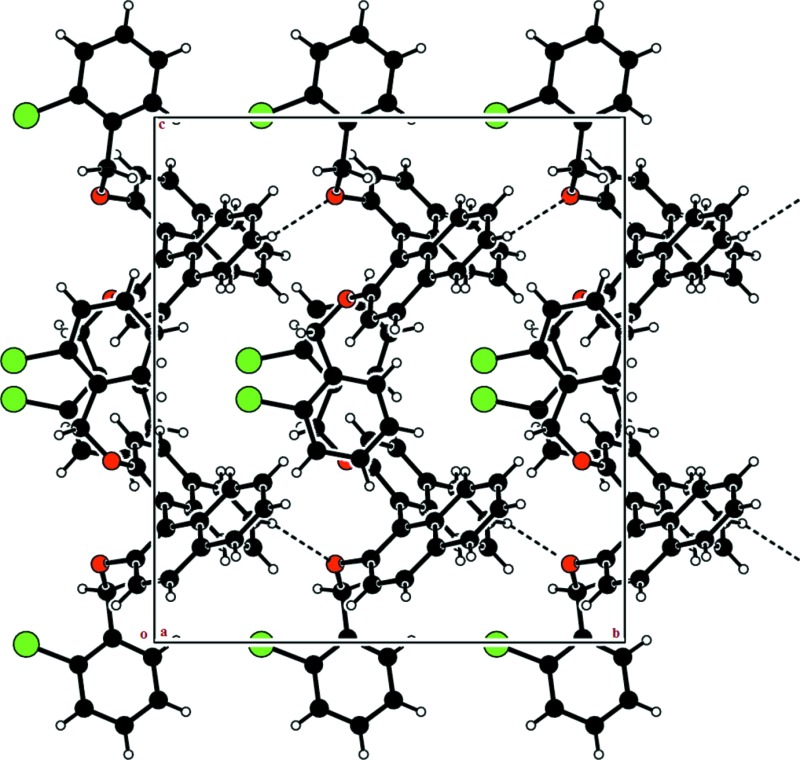
The crystal packing of the title compound, viewed along the a axis. The intermolecular interactions are shown as dashed lines (see Table 1).

. DOI: 10.1107/S2056989015014322/su5184fig1.tif
The mol­ecular structure of the title compound, with atom labelling. Displacement ellipsoids are drawn at the 30% probability level.

a . DOI: 10.1107/S2056989015014322/su5184fig2.tif
The crystal packing of the title compound, viewed along the *a* axis. The inter­molecular inter­actions are shown as dashed lines (see Table 1).

**Fig. 3.**
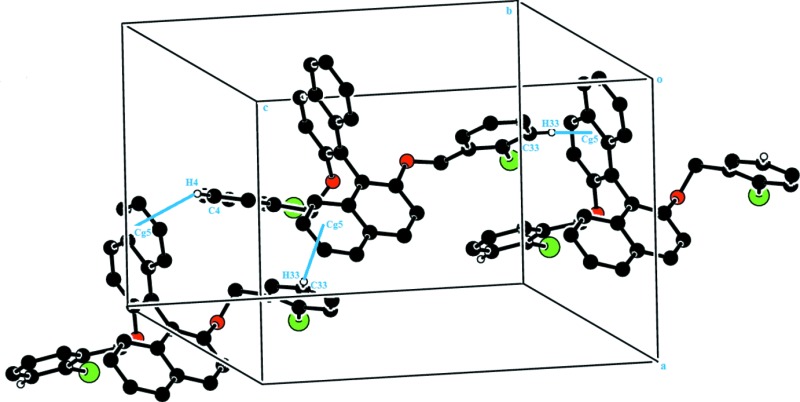
A partial view of the crystal packing of the title compound, showing the C—H···π interactions as dashed lines (see Table 1).

. DOI: 10.1107/S2056989015014322/su5184fig3.tif
A partial view of the crystal packing of the title compound, showing the C—H⋯π inter­actions as dashed lines (see Table 1).

CCDC reference: 1415827

Additional supporting information:  crystallographic information; 3D view; checkCIF report

## Figures and Tables

**Table 1:** Hydrogen-bond geometry (, ) *Cg*5 is the centroid of the C19C24 ring.

*D*H*A*	*D*H	H*A*	*D* *A*	*D*H*A*
C22H22O1^i^	0.93	2.57	3.413(4)	151
C4H4*Cg*5^ii^	0.93	2.74	3.433(4)	132
C33H33*Cg*5^iii^	0.93	2.92	3.781(6)	155

Symmetry codes: (i) 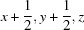; (ii) 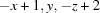; (iii) 
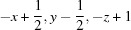
.

## supplementary crystallographic information

### S1. Comment

Naphthalene derivatives has been identified as new range of potent anti­microbials effective against a wide range of human pathogens. They occupy a central place among medicinally important compounds due to their diverse and inter­esting anti­biotic properties with minimum toxicity (Rokade & Sayyed, 2009; Upadhayaya *et al.* 2010). Herein, we report on the synthesis and crystal structure of a new bi­naphthyl derivative.

The molecular structure of the title compound is shown in Fig. 1. The chloro­phenyl ring (C1—C6) make a dihedral angle of 58.15 (12) ° with the naphthalene ring system (C8—C17), while the other chloro­phenyl ring (C29—C34) makes a dihedral angle of 76.21 (13) ° with the naphthalene ring system (C18—C27). The two naphthalene rings are inclined to one another by 76.33 (8)° and the two chloro­phenyl rings by 27.66 (16) °. Atoms O1 and O2 deviate from their respective naphthalene ring by 0.144 and 0.138 Å, respectively. The two naphthalene rings are connected at bond C17—C18, with torsion angle C19—C18—C17—C16 = 75.7 (3) °, indicating a (+) *syn*-clinal conformation for this group.

In the crystal, C—H···O hydrogen bonds link molecules into chains propagating along [110]; Table 1 and Fig. 2. The chains are linked by C—H···π inter­actions forming a three-dimensional framework (Table 1 and Fig. 3).

### S2. Synthesis and crystallization

The title compound was synthesized by reacting two equivalents of 2-chloro benzyl­bromide with one equivalent of S-BINOL in dry DMF in the presence of K_2_CO_3_ at 333 K, which successfully provided the pure title product as a colourless solid. The product was dissolved in chloro­form and heated for 2 min. The resulting solution was subjected to crystallization by slow evaporation of the solvent for 18 h resulting in the formation of single crystals.

### S3. Refinement

Crystal data, data collection and structure refinement details are summarized in Table 2. The C-bound H atoms were positioned geometrically and allowed to ride on their parent atoms: C—H = 0.93 - 0.97 Å with *U*_iso_(H) = 1.2*U*_eq_(C).

### Crystal data

**Table:**

C_34_H_24_Cl_2_O_2_	*F*(000) = 1112
*M**_r_* = 535.43	*D*_x_ = 1.333 Mg m^−^^3^
Monoclinic, *C*2	Mo *K*α radiation, λ = 0.71073 Å
Hall symbol: C 2y	Cell parameters from 3804 reflections
*a* = 11.1983 (3) Å	θ = 1.3–25.0°
*b* = 14.6094 (4) Å	µ = 0.27 mm^−^^1^
*c* = 16.3263 (4) Å	*T* = 293 K
β = 92.622 (2)°	Colourless, block
*V* = 2668.19 (12) Å^3^	0.35 × 0.30 × 0.25 mm
*Z* = 4	

### Data collection

**Table:**

Bruker SMART APEXII CCD diffractometer	4153 independent reflections
Radiation source: fine-focus sealed tube	3804 reflections with *I* > 2σ(*I*)
Graphite monochromator	*R*_int_ = 0.019
ω and φ scans	θ_max_ = 25.0°, θ_min_ = 1.3°
Absorption correction: multi-scan (*SADABS*; Bruker, 2008)	*h* = −13→13
*T*_min_ = 0.909, *T*_max_ = 0.921	*k* = −17→14
10688 measured reflections	*l* = −19→19

### Refinement

**Table:**

Refinement on *F*^2^	Secondary atom site location: difference Fourier map
Least-squares matrix: full	Hydrogen site location: inferred from neighbouring sites
*R*[*F*^2^ > 2σ(*F*^2^)] = 0.042	H-atom parameters constrained
*wR*(*F*^2^) = 0.116	*w* = 1/[σ^2^(*F*_o_^2^) + (0.0687*P*)^2^ + 1.4377*P*] where *P* = (*F*_o_^2^ + 2*F*_c_^2^)/3
*S* = 1.04	(Δ/σ)_max_ < 0.001
4153 reflections	Δρ_max_ = 0.35 e Å^−^^3^
343 parameters	Δρ_min_ = −0.47 e Å^−^^3^
1 restraint	Absolute structure: Flack (1983), 1709 (76%) Friedel pairs
Primary atom site location: structure-invariant direct methods	Absolute structure parameter: −0.01 (8)

### Special details

**Table:**

Geometry. All e.s.d.'s (except the e.s.d. in the dihedral angle between two l.s. planes) are estimated using the full covariance matrix. The cell e.s.d.'s are taken into account individually in the estimation of e.s.d.'s in distances, angles and torsion angles; correlations between e.s.d.'s in cell parameters are only used when they are defined by crystal symmetry. An approximate (isotropic) treatment of cell e.s.d.'s is used for estimating e.s.d.'s involving l.s. planes.
Refinement. Refinement of *F*^2^ against ALL reflections. The weighted *R*-factor *wR* and goodness of fit *S* are based on *F*^2^, conventional *R*-factors *R* are based on *F*, with *F* set to zero for negative *F*^2^. The threshold expression of *F*^2^ > σ(*F*^2^) is used only for calculating *R*-factors(gt) *etc*. and is not relevant to the choice of reflections for refinement. *R*-factors based on *F*^2^ are statistically about twice as large as those based on *F*, and *R*- factors based on ALL data will be even larger.

### Fractional atomic coordinates and isotropic or equivalent isotropic displacement parameters (Å^2^)

**Table:**

	*x*	*y*	*z*	*U*_iso_*/*U*_eq_	
C1	0.3136 (3)	−0.1616 (2)	1.04240 (18)	0.0530 (8)	
C2	0.2949 (3)	−0.1496 (3)	1.1254 (2)	0.0697 (10)	
H2	0.2774	−0.1995	1.1581	0.084*	
C3	0.3023 (3)	−0.0642 (3)	1.1581 (2)	0.0732 (12)	
H3	0.2899	−0.0553	1.2135	0.088*	
C4	0.3282 (3)	0.0083 (3)	1.1096 (2)	0.0662 (10)	
H4	0.3338	0.0667	1.1321	0.079*	
C5	0.3459 (2)	−0.0043 (2)	1.02794 (19)	0.0500 (7)	
H5	0.3620	0.0464	0.9958	0.060*	
C6	0.3408 (2)	−0.0892 (2)	0.99204 (15)	0.0375 (6)	
C7	0.3671 (2)	−0.1012 (2)	0.90297 (15)	0.0420 (6)	
H7A	0.4083	−0.0471	0.8845	0.050*	
H7B	0.4204	−0.1529	0.8978	0.050*	
C8	0.1865 (2)	−0.04007 (18)	0.84115 (14)	0.0317 (5)	
C9	0.0865 (2)	−0.0367 (2)	0.88989 (15)	0.0419 (6)	
H9	0.0759	−0.0815	0.9294	0.050*	
C10	0.0050 (2)	0.0319 (2)	0.87954 (17)	0.0460 (7)	
H10	−0.0599	0.0345	0.9131	0.055*	
C11	0.0178 (2)	0.0992 (2)	0.81855 (16)	0.0399 (6)	
C12	−0.0668 (3)	0.1700 (3)	0.80425 (19)	0.0544 (8)	
H12	−0.1330	0.1731	0.8364	0.065*	
C13	−0.0536 (3)	0.2330 (3)	0.7451 (2)	0.0618 (9)	
H13	−0.1108	0.2786	0.7367	0.074*	
C14	0.0464 (3)	0.2302 (2)	0.6959 (2)	0.0574 (8)	
H14	0.0555	0.2741	0.6554	0.069*	
C15	0.1301 (2)	0.1626 (2)	0.70786 (16)	0.0459 (7)	
H15	0.1953	0.1607	0.6746	0.055*	
C16	0.1197 (2)	0.09592 (18)	0.76940 (15)	0.0338 (6)	
C17	0.20583 (19)	0.02472 (17)	0.78207 (13)	0.0297 (5)	
C18	0.3126 (2)	0.02111 (17)	0.73118 (14)	0.0306 (5)	
C19	0.4102 (2)	0.08223 (17)	0.74544 (14)	0.0312 (5)	
C20	0.4120 (2)	0.14841 (19)	0.80899 (16)	0.0394 (6)	
H20	0.3495	0.1504	0.8446	0.047*	
C21	0.5042 (3)	0.2092 (2)	0.81876 (19)	0.0498 (7)	
H21	0.5031	0.2525	0.8604	0.060*	
C22	0.6005 (3)	0.2072 (2)	0.76682 (19)	0.0514 (8)	
H22	0.6619	0.2498	0.7734	0.062*	
C23	0.6040 (2)	0.1433 (2)	0.70721 (19)	0.0467 (7)	
H23	0.6694	0.1412	0.6741	0.056*	
C24	0.5096 (2)	0.07932 (18)	0.69410 (15)	0.0352 (6)	
C25	0.5086 (2)	0.0147 (2)	0.62965 (16)	0.0415 (6)	
H25	0.5737	0.0111	0.5964	0.050*	
C26	0.4138 (2)	−0.0424 (2)	0.61550 (15)	0.0405 (6)	
H26	0.4142	−0.0840	0.5725	0.049*	
C27	0.3146 (2)	−0.03851 (18)	0.66597 (14)	0.0329 (5)	
C28	0.2062 (3)	−0.15760 (19)	0.59124 (15)	0.0439 (6)	
H28A	0.1470	−0.2029	0.6049	0.053*	
H28B	0.2826	−0.1886	0.5894	0.053*	
C29	0.1738 (2)	−0.1202 (2)	0.50774 (15)	0.0439 (7)	
C30	0.1581 (3)	−0.0283 (3)	0.4912 (2)	0.0627 (9)	
H30	0.1699	0.0149	0.5326	0.075*	
C31	0.1242 (3)	−0.0006 (4)	0.4110 (3)	0.0882 (14)	
H31	0.1137	0.0613	0.3996	0.106*	
C32	0.1067 (4)	−0.0627 (5)	0.3502 (3)	0.1013 (19)	
H32	0.0845	−0.0429	0.2975	0.122*	
C33	0.1212 (4)	−0.1552 (5)	0.3653 (2)	0.0944 (17)	
H33	0.1088	−0.1978	0.3234	0.113*	
C34	0.1541 (3)	−0.1829 (3)	0.44307 (18)	0.0650 (10)	
O1	0.26269 (16)	−0.11565 (12)	0.85074 (10)	0.0398 (4)	
O2	0.21372 (16)	−0.09115 (14)	0.65440 (10)	0.0453 (5)	
Cl1	0.30029 (15)	−0.27265 (8)	1.00356 (7)	0.1030 (4)	
Cl2	0.16806 (12)	−0.29928 (8)	0.46325 (7)	0.0972 (4)	

### Atomic displacement parameters (Å^2^)

**Table:**

	*U*^11^	*U*^22^	*U*^33^	*U*^12^	*U*^13^	*U*^23^
C1	0.0660 (18)	0.048 (2)	0.0444 (16)	−0.0107 (15)	−0.0038 (13)	0.0004 (14)
C2	0.076 (2)	0.091 (3)	0.0419 (17)	−0.019 (2)	0.0022 (15)	0.0106 (19)
C3	0.061 (2)	0.113 (4)	0.0459 (18)	−0.014 (2)	0.0050 (14)	−0.023 (2)
C4	0.0537 (17)	0.075 (3)	0.069 (2)	0.0005 (18)	−0.0026 (15)	−0.036 (2)
C5	0.0454 (15)	0.0470 (19)	0.0571 (17)	0.0010 (13)	−0.0038 (12)	−0.0084 (15)
C6	0.0331 (12)	0.0399 (16)	0.0389 (13)	0.0002 (11)	−0.0036 (9)	−0.0021 (12)
C7	0.0380 (13)	0.0483 (17)	0.0395 (13)	0.0013 (12)	−0.0008 (10)	0.0018 (13)
C8	0.0338 (12)	0.0331 (14)	0.0278 (11)	−0.0012 (10)	−0.0017 (9)	−0.0067 (11)
C9	0.0405 (14)	0.0537 (18)	0.0318 (12)	−0.0075 (13)	0.0040 (10)	0.0044 (13)
C10	0.0367 (13)	0.062 (2)	0.0399 (14)	−0.0019 (13)	0.0095 (11)	−0.0062 (14)
C11	0.0352 (12)	0.0509 (18)	0.0335 (13)	0.0045 (12)	−0.0001 (10)	−0.0082 (13)
C12	0.0400 (14)	0.070 (2)	0.0526 (17)	0.0163 (15)	0.0014 (12)	−0.0112 (18)
C13	0.0600 (19)	0.065 (2)	0.0600 (19)	0.0313 (17)	−0.0033 (14)	−0.0035 (18)
C14	0.0631 (19)	0.053 (2)	0.0561 (17)	0.0161 (16)	−0.0010 (14)	0.0084 (17)
C15	0.0478 (15)	0.0485 (17)	0.0416 (14)	0.0065 (13)	0.0038 (11)	0.0011 (14)
C16	0.0337 (12)	0.0377 (15)	0.0298 (12)	0.0013 (11)	−0.0016 (9)	−0.0073 (11)
C17	0.0312 (11)	0.0330 (14)	0.0246 (11)	−0.0028 (10)	−0.0012 (9)	−0.0065 (10)
C18	0.0340 (11)	0.0318 (14)	0.0259 (11)	0.0039 (10)	0.0008 (9)	0.0012 (10)
C19	0.0314 (11)	0.0304 (13)	0.0314 (12)	0.0008 (10)	−0.0014 (9)	0.0079 (11)
C20	0.0393 (13)	0.0381 (16)	0.0406 (13)	0.0018 (12)	0.0000 (10)	−0.0040 (12)
C21	0.0559 (17)	0.0396 (18)	0.0530 (16)	−0.0059 (13)	−0.0072 (13)	−0.0036 (14)
C22	0.0457 (15)	0.0444 (19)	0.0631 (18)	−0.0149 (13)	−0.0096 (13)	0.0092 (16)
C23	0.0364 (14)	0.0491 (18)	0.0545 (17)	−0.0068 (12)	0.0020 (11)	0.0156 (15)
C24	0.0360 (12)	0.0356 (14)	0.0340 (12)	0.0019 (11)	0.0008 (10)	0.0083 (11)
C25	0.0388 (13)	0.0454 (16)	0.0413 (14)	0.0044 (12)	0.0115 (10)	0.0063 (13)
C26	0.0523 (15)	0.0406 (16)	0.0291 (12)	0.0056 (13)	0.0073 (11)	−0.0045 (12)
C27	0.0373 (12)	0.0325 (14)	0.0288 (11)	−0.0028 (11)	0.0003 (9)	−0.0009 (11)
C28	0.0578 (16)	0.0390 (17)	0.0348 (13)	−0.0113 (13)	0.0007 (11)	−0.0080 (12)
C29	0.0383 (13)	0.059 (2)	0.0348 (13)	−0.0060 (12)	0.0038 (10)	−0.0006 (13)
C30	0.0557 (18)	0.068 (3)	0.064 (2)	0.0025 (16)	0.0019 (14)	0.0148 (19)
C31	0.069 (2)	0.108 (4)	0.088 (3)	0.017 (2)	0.007 (2)	0.043 (3)
C32	0.068 (2)	0.186 (6)	0.050 (2)	0.020 (3)	0.0014 (17)	0.028 (3)
C33	0.075 (2)	0.172 (6)	0.0352 (18)	−0.004 (3)	−0.0038 (16)	−0.014 (3)
C34	0.0510 (17)	0.105 (3)	0.0391 (15)	−0.0080 (18)	0.0078 (13)	−0.0074 (19)
O1	0.0474 (10)	0.0351 (11)	0.0365 (9)	0.0011 (8)	−0.0036 (7)	−0.0017 (8)
O2	0.0516 (10)	0.0506 (12)	0.0342 (9)	−0.0143 (9)	0.0077 (7)	−0.0180 (9)
Cl1	0.1870 (13)	0.0508 (6)	0.0725 (6)	−0.0226 (7)	0.0191 (7)	0.0005 (5)
Cl2	0.1348 (10)	0.0795 (8)	0.0790 (6)	−0.0275 (6)	0.0244 (6)	−0.0426 (6)

### Geometric parameters (Å, º)

**Table:**

C1—C6	1.382 (4)	C18—C27	1.377 (3)
C1—C2	1.392 (4)	C18—C19	1.422 (3)
C1—Cl1	1.746 (3)	C19—C20	1.418 (4)
C2—C3	1.357 (6)	C19—C24	1.424 (3)
C2—H2	0.9300	C20—C21	1.365 (4)
C3—C4	1.362 (6)	C20—H20	0.9300
C3—H3	0.9300	C21—C22	1.403 (4)
C4—C5	1.370 (5)	C21—H21	0.9300
C4—H4	0.9300	C22—C23	1.350 (4)
C5—C6	1.372 (4)	C22—H22	0.9300
C5—H5	0.9300	C23—C24	1.421 (4)
C6—C7	1.507 (3)	C23—H23	0.9300
C7—O1	1.431 (3)	C24—C25	1.413 (4)
C7—H7A	0.9700	C25—C26	1.361 (4)
C7—H7B	0.9700	C25—H25	0.9300
C8—C17	1.376 (3)	C26—C27	1.414 (3)
C8—O1	1.400 (3)	C26—H26	0.9300
C8—C9	1.404 (3)	C27—O2	1.373 (3)
C9—C10	1.361 (4)	C28—O2	1.416 (3)
C9—H9	0.9300	C28—C29	1.498 (4)
C10—C11	1.411 (4)	C28—H28A	0.9700
C10—H10	0.9300	C28—H28B	0.9700
C11—C12	1.414 (4)	C29—C30	1.379 (5)
C11—C16	1.426 (3)	C29—C34	1.408 (5)
C12—C13	1.348 (5)	C30—C31	1.405 (6)
C12—H12	0.9300	C30—H30	0.9300
C13—C14	1.408 (4)	C31—C32	1.353 (8)
C13—H13	0.9300	C31—H31	0.9300
C14—C15	1.369 (4)	C32—C33	1.382 (8)
C14—H14	0.9300	C32—H32	0.9300
C15—C16	1.408 (4)	C33—C34	1.367 (6)
C15—H15	0.9300	C33—H33	0.9300
C16—C17	1.427 (3)	C34—Cl2	1.737 (4)
C17—C18	1.488 (3)		
			
C6—C1—C2	122.0 (3)	C27—C18—C17	119.7 (2)
C6—C1—Cl1	120.8 (2)	C19—C18—C17	121.2 (2)
C2—C1—Cl1	117.2 (3)	C20—C19—C18	122.2 (2)
C3—C2—C1	119.4 (4)	C20—C19—C24	117.7 (2)
C3—C2—H2	120.3	C18—C19—C24	120.0 (2)
C1—C2—H2	120.3	C21—C20—C19	121.0 (2)
C2—C3—C4	119.7 (3)	C21—C20—H20	119.5
C2—C3—H3	120.1	C19—C20—H20	119.5
C4—C3—H3	120.1	C20—C21—C22	120.9 (3)
C3—C4—C5	120.4 (4)	C20—C21—H21	119.5
C3—C4—H4	119.8	C22—C21—H21	119.5
C5—C4—H4	119.8	C23—C22—C21	119.9 (3)
C4—C5—C6	122.1 (3)	C23—C22—H22	120.0
C4—C5—H5	118.9	C21—C22—H22	120.0
C6—C5—H5	118.9	C22—C23—C24	121.2 (3)
C5—C6—C1	116.3 (2)	C22—C23—H23	119.4
C5—C6—C7	120.7 (3)	C24—C23—H23	119.4
C1—C6—C7	122.9 (3)	C25—C24—C23	122.2 (2)
O1—C7—C6	113.71 (19)	C25—C24—C19	118.6 (2)
O1—C7—H7A	108.8	C23—C24—C19	119.2 (2)
C6—C7—H7A	108.8	C26—C25—C24	121.0 (2)
O1—C7—H7B	108.8	C26—C25—H25	119.5
C6—C7—H7B	108.8	C24—C25—H25	119.5
H7A—C7—H7B	107.7	C25—C26—C27	120.3 (2)
C17—C8—O1	120.42 (19)	C25—C26—H26	119.9
C17—C8—C9	121.9 (2)	C27—C26—H26	119.9
O1—C8—C9	117.5 (2)	O2—C27—C18	114.76 (19)
C10—C9—C8	120.2 (2)	O2—C27—C26	124.1 (2)
C10—C9—H9	119.9	C18—C27—C26	121.1 (2)
C8—C9—H9	119.9	O2—C28—C29	114.6 (2)
C9—C10—C11	120.8 (2)	O2—C28—H28A	108.6
C9—C10—H10	119.6	C29—C28—H28A	108.6
C11—C10—H10	119.6	O2—C28—H28B	108.6
C10—C11—C12	122.5 (2)	C29—C28—H28B	108.6
C10—C11—C16	118.9 (2)	H28A—C28—H28B	107.6
C12—C11—C16	118.6 (3)	C30—C29—C34	118.2 (3)
C13—C12—C11	121.6 (3)	C30—C29—C28	123.9 (3)
C13—C12—H12	119.2	C34—C29—C28	117.9 (3)
C11—C12—H12	119.2	C29—C30—C31	119.3 (4)
C12—C13—C14	120.3 (3)	C29—C30—H30	120.3
C12—C13—H13	119.8	C31—C30—H30	120.3
C14—C13—H13	119.8	C32—C31—C30	120.9 (5)
C15—C14—C13	119.8 (3)	C32—C31—H31	119.5
C15—C14—H14	120.1	C30—C31—H31	119.5
C13—C14—H14	120.1	C31—C32—C33	120.9 (4)
C14—C15—C16	121.5 (3)	C31—C32—H32	119.6
C14—C15—H15	119.3	C33—C32—H32	119.6
C16—C15—H15	119.3	C34—C33—C32	118.7 (5)
C15—C16—C11	118.2 (2)	C34—C33—H33	120.7
C15—C16—C17	122.0 (2)	C32—C33—H33	120.7
C11—C16—C17	119.7 (2)	C33—C34—C29	122.0 (5)
C8—C17—C16	118.4 (2)	C33—C34—Cl2	118.9 (4)
C8—C17—C18	121.6 (2)	C29—C34—Cl2	119.0 (2)
C16—C17—C18	120.0 (2)	C8—O1—C7	115.3 (2)
C27—C18—C19	119.0 (2)	C27—O2—C28	120.38 (19)
			
C6—C1—C2—C3	0.6 (5)	C17—C18—C19—C20	1.0 (4)
Cl1—C1—C2—C3	−179.0 (3)	C27—C18—C19—C24	−1.8 (3)
C1—C2—C3—C4	−0.1 (5)	C17—C18—C19—C24	−177.9 (2)
C2—C3—C4—C5	0.4 (5)	C18—C19—C20—C21	−176.8 (3)
C3—C4—C5—C6	−1.2 (5)	C24—C19—C20—C21	2.1 (4)
C4—C5—C6—C1	1.6 (4)	C19—C20—C21—C22	−0.8 (4)
C4—C5—C6—C7	−176.8 (3)	C20—C21—C22—C23	−1.3 (4)
C2—C1—C6—C5	−1.3 (4)	C21—C22—C23—C24	2.0 (4)
Cl1—C1—C6—C5	178.3 (2)	C22—C23—C24—C25	176.9 (3)
C2—C1—C6—C7	177.0 (3)	C22—C23—C24—C19	−0.6 (4)
Cl1—C1—C6—C7	−3.4 (4)	C20—C19—C24—C25	−179.1 (2)
C5—C6—C7—O1	−104.8 (3)	C18—C19—C24—C25	−0.2 (4)
C1—C6—C7—O1	77.0 (3)	C20—C19—C24—C23	−1.4 (3)
C17—C8—C9—C10	0.0 (4)	C18—C19—C24—C23	177.5 (2)
O1—C8—C9—C10	−175.7 (2)	C23—C24—C25—C26	−176.2 (3)
C8—C9—C10—C11	1.7 (4)	C19—C24—C25—C26	1.4 (4)
C9—C10—C11—C12	178.1 (3)	C24—C25—C26—C27	−0.7 (4)
C9—C10—C11—C16	−1.9 (4)	C19—C18—C27—O2	−176.5 (2)
C10—C11—C12—C13	−179.2 (3)	C17—C18—C27—O2	−0.4 (3)
C16—C11—C12—C13	0.8 (4)	C19—C18—C27—C26	2.7 (4)
C11—C12—C13—C14	−0.4 (5)	C17—C18—C27—C26	178.8 (2)
C12—C13—C14—C15	0.5 (5)	C25—C26—C27—O2	177.7 (2)
C13—C14—C15—C16	−0.9 (5)	C25—C26—C27—C18	−1.4 (4)
C14—C15—C16—C11	1.2 (4)	O2—C28—C29—C30	3.2 (4)
C14—C15—C16—C17	179.5 (3)	O2—C28—C29—C34	−174.4 (2)
C10—C11—C16—C15	178.9 (3)	C34—C29—C30—C31	−0.5 (4)
C12—C11—C16—C15	−1.1 (4)	C28—C29—C30—C31	−178.1 (3)
C10—C11—C16—C17	0.6 (4)	C29—C30—C31—C32	0.1 (5)
C12—C11—C16—C17	−179.4 (2)	C30—C31—C32—C33	0.2 (6)
O1—C8—C17—C16	174.3 (2)	C31—C32—C33—C34	−0.2 (6)
C9—C8—C17—C16	−1.4 (3)	C32—C33—C34—C29	−0.2 (6)
O1—C8—C17—C18	−4.9 (3)	C32—C33—C34—Cl2	178.0 (3)
C9—C8—C17—C18	179.4 (2)	C30—C29—C34—C33	0.5 (4)
C15—C16—C17—C8	−177.2 (2)	C28—C29—C34—C33	178.3 (3)
C11—C16—C17—C8	1.0 (3)	C30—C29—C34—Cl2	−177.7 (2)
C15—C16—C17—C18	2.0 (3)	C28—C29—C34—Cl2	0.1 (3)
C11—C16—C17—C18	−179.8 (2)	C17—C8—O1—C7	86.3 (3)
C8—C17—C18—C27	78.8 (3)	C9—C8—O1—C7	−97.9 (3)
C16—C17—C18—C27	−100.4 (3)	C6—C7—O1—C8	68.7 (3)
C8—C17—C18—C19	−105.2 (3)	C18—C27—O2—C28	−177.7 (2)
C16—C17—C18—C19	75.6 (3)	C26—C27—O2—C28	3.2 (4)
C27—C18—C19—C20	177.0 (2)	C29—C28—O2—C27	−80.7 (3)

### Hydrogen-bond geometry (Å, º)

Cg5 is the centroid of the C19–C24 ring.

**Table:**

*D*—H···*A*	*D*—H	H···*A*	*D*···*A*	*D*—H···*A*
C22—H22···O1^i^	0.93	2.57	3.413 (4)	151
C4—H4···*Cg*5^ii^	0.93	2.74	3.433 (4)	132
C33—H33···*Cg*5^iii^	0.93	2.92	3.781 (6)	155

Symmetry codes: (i) *x*+1/2, *y*+1/2, *z*; (ii) −*x*+1, *y*, −*z*+2; (iii) −*x*+1/2, *y*−1/2, −*z*+1.
