# Supplementary figures and images for: Crystal structure of 2,2′-bis­[(2-chloro­benz­yl)­oxy]-1,1′-bi­naphthalene
Source: Acta Crystallogr E Crystallogr Commun. 2015 Aug 6;71(Pt 9):o637–8. doi: 10.1107/S2056989015014322 (PMC4555433; doi:10.1107/S2056989015014322)

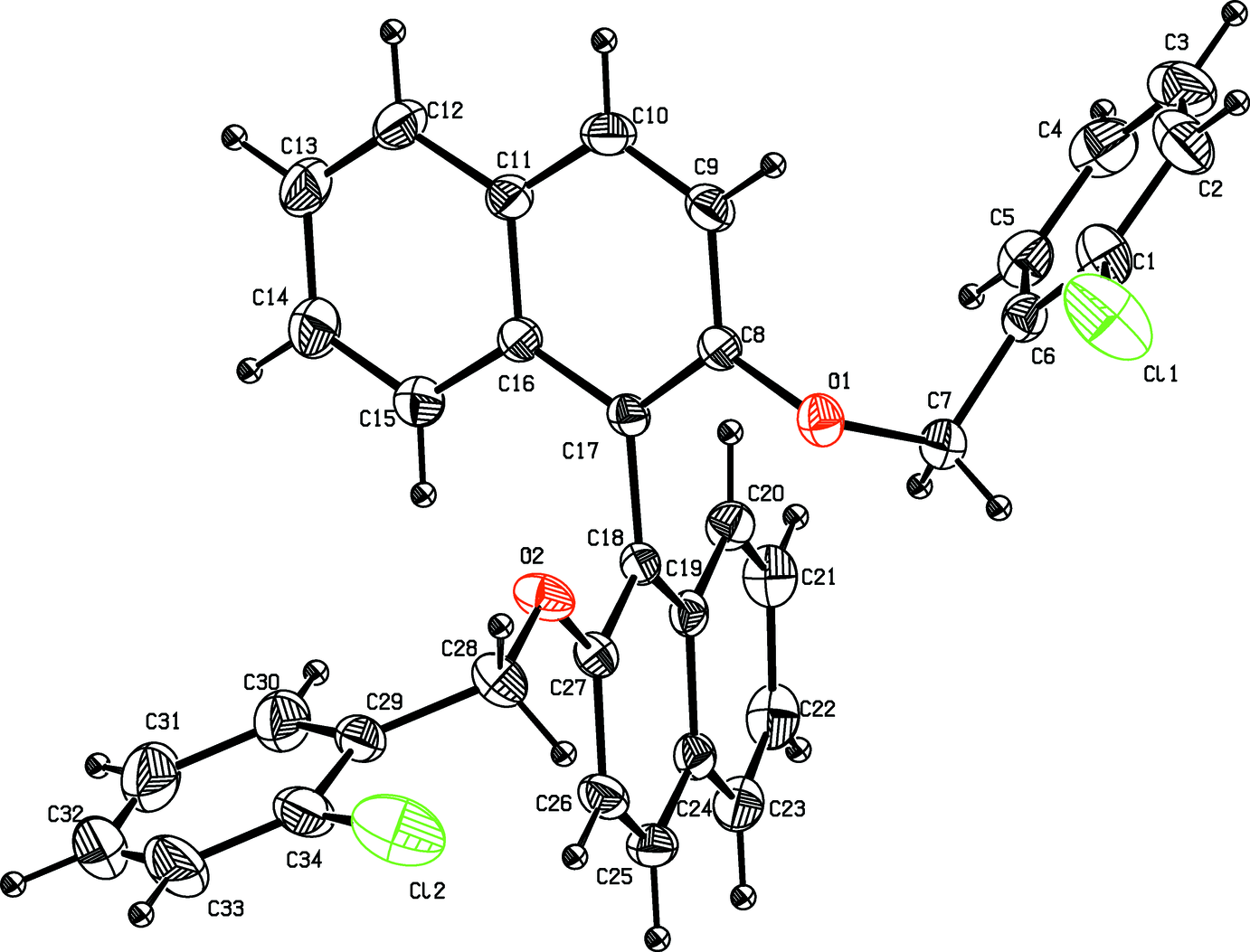

Supplement: Supplementary file 4 [file e-71-0o637-fig1.tif]

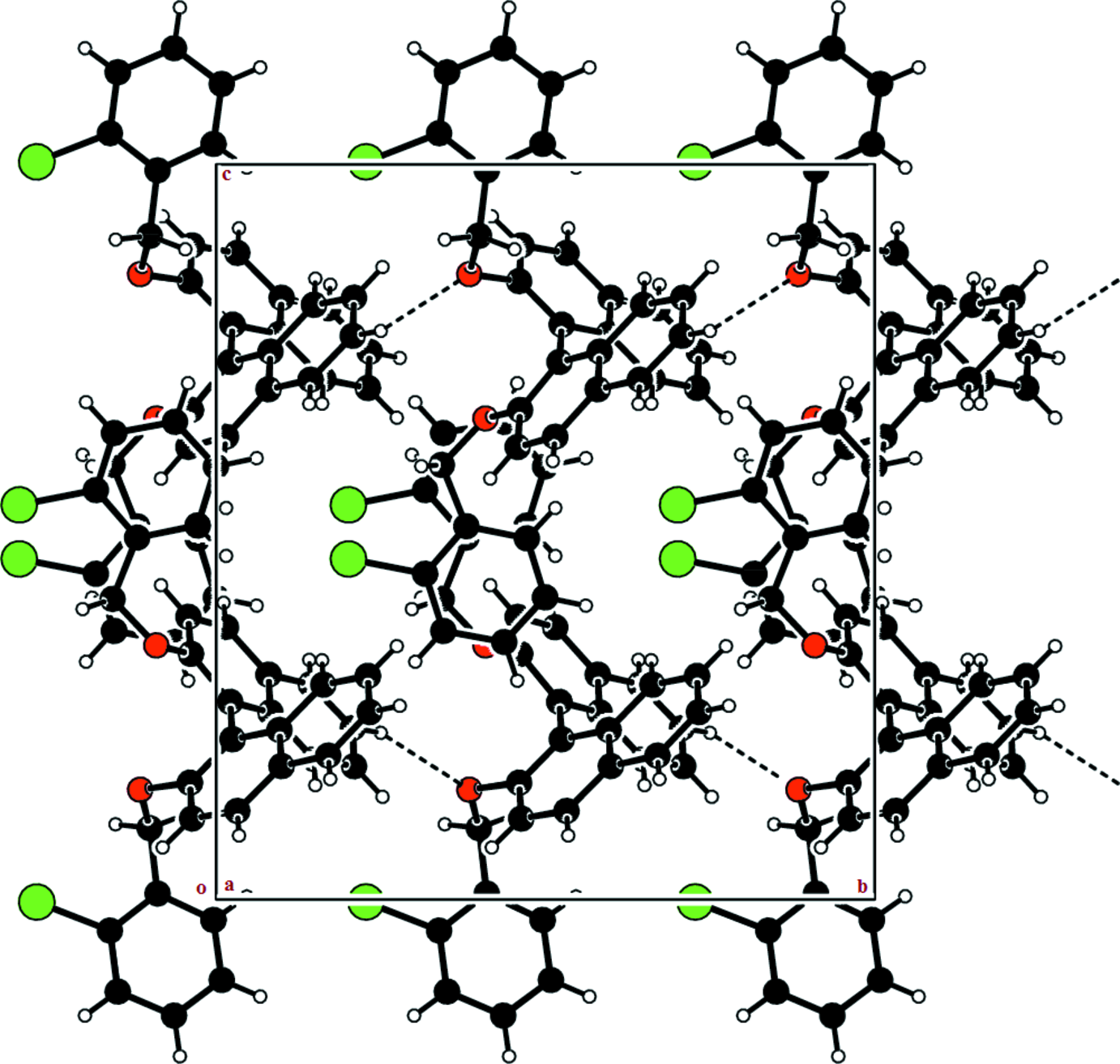

Supplement: Supplementary file 5 [file e-71-0o637-fig2.tif]

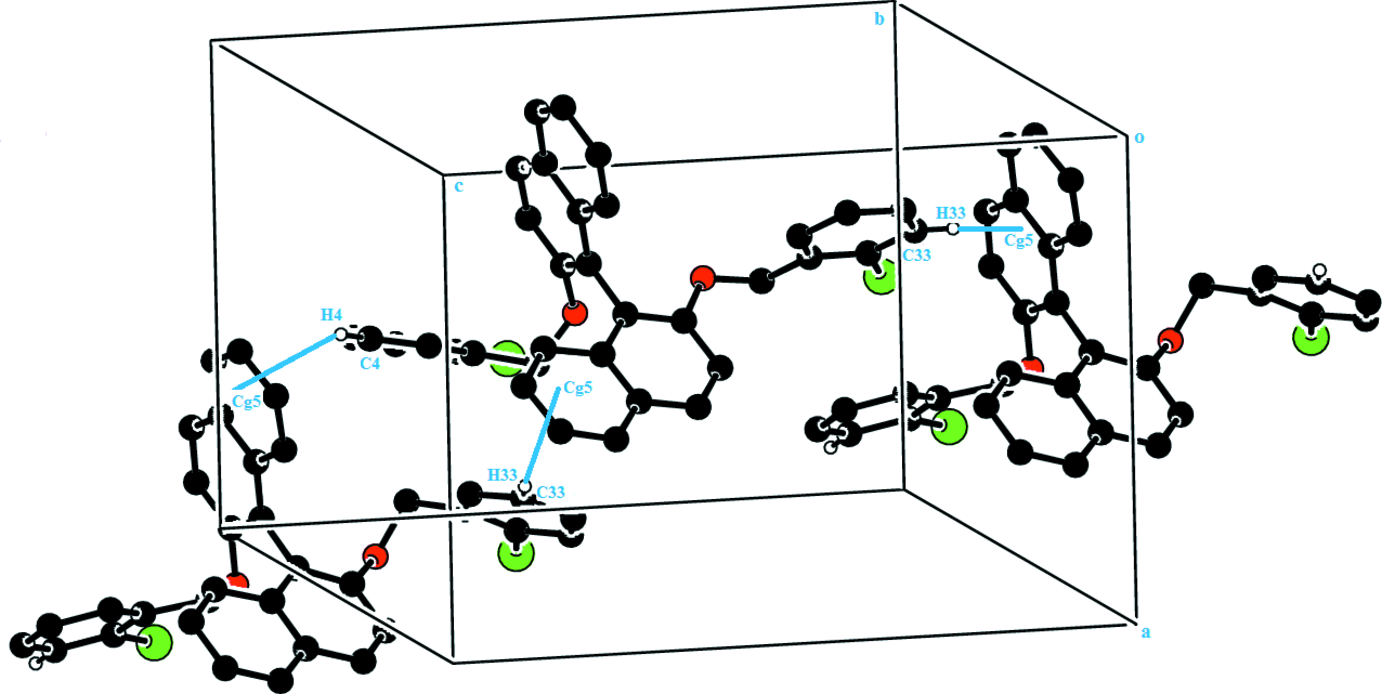

Supplement: Supplementary file 6 [file e-71-0o637-fig3.tif]
